# Supplementary material for: How exclusion criteria can hinder eligibility for lung cancer studies among different racial and ethnic groups
Source: PLOS Digit Health. 2026 Mar 10;5(3):e0001262. doi: 10.1371/journal.pdig.0001262 (PMC12974856; doi:10.1371/journal.pdig.0001262)
Supplement: S1 File — Detailed description of text analysis process. (DOCX) [file pdig.0001262.s001.docx]

S1 eAppendix

**Text Analysis Process**

On the ClinicalTrial.gov website, eligibility criteria were typically stored as a large text – usually under a bulleted or numbered list under “Inclusion Criteria,” followed by a similar bulleted or numbered list for “Exclusion Criteria.” Text that occurred after “Exclusion Criteria” but before “Inclusion Criteria” were extracted. The exclusion criteria were then split into separate entries where there was a return line.

**Classifying General Exclusion Criteria Categories**

The categories for “Inability to Consent” and “Provider bias” both captured exclusion criteria that relied on the judgement of the provider or investigator. These categories were created in consultation with an addiction medicine physician who explained the different assessments physicians perform to evaluate a patient’s ability to provide medical consent, particularly when handling patients who are cognitively impaired or are under the influence drugs. “Inability to Consent” captured exclusion criteria that included specific situations where a patient was clinically deemed unfit to participate in a clinical trial study.^[[1]](#footnote-1)^ These included examples such as, “prior or ongoing clinically relevant illness, medical condition, surgical history, physical finding, or laboratory abnormality that, in the investigator's opinion, could affect the safety of the participant.” Other examples included psychiatric illnesses, social situations (i.e., incarceration), or mobility issues (i.e., increased fall risk) that in the opinion of the investigator would severely limit the patient’s compliance with the study requirement.

Criteria classified under “Gatekeeper Bias” were also based on the judgement of the clinician; however, unlike the criteria classified under “Inability to Consent,” the exclusion criteria did not include specific details on what would disqualify a patient (i.e., patient is cognitively impaired) and used general language that was less reliant on specific clinical assessments and hence more likely to introduce bias – conscious or unconscious- into the decision-making process. Examples of such statements included: “any condition which in the investigator’s opinion deems the participant an unsuitable candidate.”

**Search Terms used to capture lung cancer studies on ClinicalTrials.gov**

{Small-cell lung cancer, metastatic NSCLS, non-small cell lung cancer, lung neoplasm}

**Categorizing criteria into their respective categories**

The following describes the reason why 16% of criteria were categorized into more than one category and how we handled such criteria with more than one category. To illustrate, exclusion criteria #15 from study [NCT04434482](https://clinicaltrials.gov/study/NCT04434482?term=NCT04434482&rank=1) reads:

Patients who have major cardiovascular diseases (such as congestive heart failure, unstable angina, atrial fibrillation, arrhythmia); patients who have acute myocardial infarction, unstable angina, stroke, or transient ischemic attack within 6 months prior to the first dose of the ips; patients who have congestive heart failure (≥new york heart association \[nyha\] classification class ii); patients who have severe arrhythmia requiring medication (including qt interval \[qtc\] prolongation corrected by the fridericia's formula \[qtcf\] of more than 480 msec, pacemaker installation, and previous diagnosis of congenital long qt syndrome).

The last criterion – “severe arrhythmia requiring medication including qt interval prolongation corrected by the fridericias’ formula of more than 480 msec” was categorized as both medical condition and clinical exclusion. The research team underwent 3 iterations until the changes to regular expression patterns led to an incremental decrease in the percent of criteria that was doubly categorized. We completed this process after 3 iterations due to diminishing returns associated with each iteration, such that each time we updated the regular expression (regex) pattern to match a target string to account for a specific criteria, (i.e., the pattern “cit” was modified to match “cit” *not* preceded by “prior”, the categorization for certain cases such as like “any history of immune-mediated grade 3 adverse event prior to cit”) would improve, but previously correctly categorized criteria would no longer be correctly categorized (i.e., “has received prior radiotherapy”), creating a type of iterative regression effect where fixing one set of mismatches introduced new mismatches in previously correct cases. The categorization efforts were then halted to ensure correct categorization across most criteria.

1. Clinicians typically use a combination of objective and subjective methods when evaluating the mental fitness of patients. Objective criteria rely on assessments, such as the patients’ vitals (i.e., temperature, blood pressure, abdominal exams); urinalysis; and pupil dilation; whereas more subjective criteria use assessments that evaluate the patient’s orientation status (alertness and orientation to person/place/time/situation); recall ability; and emotional and mental well-being based on questions, such as “Let’s say this clinic is on fire. What would be the first thing you do?”). After a series of assessments, clinicians will determine whether a patient is able to consent to treatment. Criteria that referenced specific parameters that clinicians could use to determine a patient’s ability to consent were classified under “inability to consent.” [↑](#footnote-ref-1)
