# Supplementary material for: How exclusion criteria can hinder eligibility for lung cancer studies among different racial and ethnic groups
Source: PLOS Digit Health. 2026 Mar 10;5(3):e0001262. doi: 10.1371/journal.pdig.0001262 (PMC12974856; doi:10.1371/journal.pdig.0001262)
Supplement: Attachment — Submitted filename: Response Letter.docx [file pdig.0001262.s003.docx]

January 12, 2026

Dear Editors of *PLOS Digital Health*

Thank you for the opportunity to revise our manuscript, entitled “How Exclusion Criteria Can Hinder Eligibility for Lung Cancer Studies Among Different Racial and Ethnic Groups.” We appreciate the time and effort that the editors and reviewers devoted to evaluating our work and are grateful for their constructive feedback.

Below, we provide a point-by-point response to each of the reviewer’s feedback. The reviewer’s feedback is single spaced, and our response is double-spaced and indented for easier viewing.

Reviewer #1:

Firstly, the manuscript notes that approximately 16% of exclusion criteria were “compounded” (classified into multiple categories), but it fails to provide details on how these compound criteria were analyzed. For instance, were trials with compound criteria weighted differently? Or were patients excluded if they met any of the multiple criteria categories?

This is a good question. Patients were excluded if they met any of the multiple criteria categories. Trials with compound criteria were not weighted differently. If a patient met any of the medical history / condition criteria, then they were excluded. A sentence that more explicitly explains how compounded criteria was handled has been added on page 7.

Secondly, Black (3.3%) and Hispanic/Latino (1.8%) patients make up a small fraction of the study sample. Although the authors acknowledge this as a limitation, they do not report a priori power calculations to confirm whether the sample size was sufficient to detect meaningful differences in exclusion rates for these groups. For example, this could include potential associations for Hispanic/Latino patients.

We appreciate the reviewer's important point regarding power calculations. Prior to data collection, we conducted an a priori power analysis to determine the minimum sample size required for the logistic regression analysis. Based on 6 predictors (race/ethnicity, gender, and income), a small effect size and power of .95, the analysis indicated a minimum required sample of n=395. To account for potential or incomplete data, an additional 25 participants were added, yielding a minimum size of n=420. We acknowledge that this power calculation assumes equal distribution across racial groups; however, unequal representation across groups may result in reduced power to detect differences for smaller racial subgroups. Indeed, our actual patient population was 4,096, well above the minimum size of n=420. However, the racial and ethnic groups were unbalanced. To address the underlying concern about sample size adequacy, we have performed a **retrospective sensitivity analysis to** determine the minimum detectable effect (MDE) for each racial group. This analysis demonstrates that our sample sizes provided adequate power to detect medium disparities (≥10 percentage points) for AAPI patients (n=512), moderate power for Black patients (n=132), but insufficient power for Latino patients (n=72). Specifically, the minimum detectable effects were:

- AAPI: 6.4 percentage points (OR≈1.38)
- Black: 11.8 percentage points (OR≈1.73)
- Latino: 15.8 percentage points (OR≈2.36)

This reveals that the non-significant finding for Latino patients may reflect Type II error rather than true absence of disparity - a limitation we have now explicitly acknowledged in our revised manuscript. We have added a subsection to our Methods describing this sensitivity analysis (p. 8) and clarified that it represents a retrospective assessment rather than prospective planning. We address this also in the results (p.10) and discussion sections (p. 12-13, 15).

Thirdly, Table 3 lists the top comorbidities by race/ethnicity but does not include prevalence data for these conditions. For example, it does not show whether Type 2 diabetes is significantly more common in AAPI patients than in White patients. Without quantifying the differences in comorbidity prevalence, the causal link between comorbidities and exclusion risk remains indirect. We suggest supplementing Table 3 with comorbidity prevalence rates (and 95% confidence intervals).

Thank you for pointing this out. Since the table shows frequency based on the comorbidities across all racial groups, we revised Table 3’s heading to “Top Medical Comorbidities Among Race and Ethnic Groups and Their Status as Common Exclusion Criteria.”

Fourthly, the authors note that clinical exclusion criteria (e.g., laboratory values like neutrophil counts) were not included in the analysis, but they do not elaborate on their potential impact on minority groups. For instance, benign ethnic neutropenia, which is common in Black patients, is cited in references 24–25 but is not linked to hypothetical exclusion rates. We recommend adding a paragraph to the “Discussion” section estimating how including these criteria might amplify racial disparities.

Thank you for raising this important point. In response, we have expanded the discussion to acknowledge this limitation and cite recent evidence (p. 13-14)

Fifthly, there is a critical discrepancy between the title and content of Table 3. The title reads “Types of Exclusion Criteria in Lung Cancer Trials,” but the table actually presents “Top Medical Comorbidities by Race and Ethnic Group.” This error will confuse readers and must be corrected. For example, the title could be revised to “Top Medical Comorbidities by Race and Ethnic Group, and Their Status as Common Exclusion Criteria.” Additionally, ensure that column headers are consistent. For instance, the third column lacks a clear header; it should be “Listed as a Common Exclusion Criterion in Lung Cancer Studies.”

Thank you for pointing this out. We’ve revised the title of Table 4 (Previously Table 3) to “Top Medical Comorbidities by Race and Ethnic Group and their Status as Common Exclusion Criteria” as you suggested. We also clarified the third header based on your suggestion.

Finally, the authors attribute non-significant results for Hispanic/Latino patients to “sample heterogeneity” but do not explore potential subgroup differences. For example, Mexican American and Puerto Rican patients may have distinct comorbidity profiles. If the EMR data includes subgroup identifiers, adding a post-hoc subgroup analysis of exclusion rates by Hispanic/Latino ethnicity would provide deeper insights into disparities. If not, the “Discussion” section should cite literature on Hispanic/Latino comorbidity heterogeneity to contextualize the null finding.

We appreciate the reviewer’s important point regarding heterogeneity within Hispanic/Latino populations. We agree that subgroups such as Mexican American and Puerto Rican patients may have distinct comorbidity profiles and exclusion risks. Unfortunately, the EMR data used in this study did not include sufficiently granular Hispanic/Latino subgroup identifiers to support a reliable post-hoc subgroup analysis. We have therefore revised the Discussion (p.12) to explicitly acknowledge this limitation and to contextualize the null findings using prior literature documenting substantial heterogeneity in comorbidity burden and chronic disease prevalence across Hispanic/Latino subgroups. Specifically, we now cite studies demonstrating differential patterns of comorbidities across Hispanic/Latino ethnicities, which may be obscured when treating this population as a single analytic category. We note that such heterogeneity may have attenuated observed associations in our analysis and highlight the need for future studies with more granular ethnicity data.

Reviewer #2:

This study addresses an important and timely question: whether clinical trial exclusion criteria disproportionately affect racial and ethnic minority patients with lung cancer. The triangulation of ClinicalTrials.gov data with EMR records is a creative approach, and the topic aligns well with ongoing efforts to improve diversity in clinical research. I appreciate the authors' effort to include AAPI populations, who are often overlooked in health disparities research.

However, I have identified several concerns that require substantial revision before this manuscript is suitable for publication.

Thank you for your encouraging feedback.

The central claim of the paper is that minority patients are excluded from trials because of their comorbidities. However, this mechanism is assumed rather than demonstrated. The authors do not report comorbidity prevalence by race/ethnicity within their own sample, nor do they show that racial differences in exclusion are mediated by comorbidity burden. Table 3 lists common comorbidities per group but provides no rates, counts, or statistical comparisons. Without this evidence, the association between race and exclusion could reflect unmeasured confounding, site-specific coding practices, or other artifacts. A mediation analysis or at minimum a descriptive table showing differential comorbidity burden by race is essential to support the paper's core argument.

Thank you for your feedback. We have included prevalence data on some of the comorbidities listed in the chart by race/ethnicity from existing literature. However, prevalence data by race for certain conditions, such as GERD, lung disease, or pulmonary disease, were unavailable. For GERD, only the percentage of patients reporting GERD symptoms is available, with no confidence intervals reported. For liver disease, race-specific data are available only as age-adjusted percentages with standard errors, rather than prevalence estimates with confidence intervals. We changed the name of type 2 diabetes to diagnosed diabetes to align with available prevalence data, as type-specific estimates are not consistently reported by race. We also changed primary hypertension to hypertension to better align with the available prevalence data. Finally, lung disease and pulmonary disease are broad categories for which no race-specific prevalence estimates exist; available data are typically limited to specific conditions such as COPD.

The small sample sizes for Black (3.3%) and Latino (1.8%) patients are acknowledged only briefly at the end of the Discussion. Given that these are central to the study's conclusions, this limitation requires more prominent treatment and should temper the strength of the claims made throughout.

We appreciate your feedback. We’ve addressed this concern by including information about the a priori power analysis that was conducted prior to data collection, in addition to a sensitivity analysis that was done post-hoc. We also more prominently note the limitations related to the underpowered Latino sample. Please refer to our response to Reviewer #1 on p. 2 of this Letter for additional details.

The handling of non-significant findings for Latino patients is problematic. The authors speculate that heterogeneity may be "masking potential associations," but this is post-hoc reasoning without empirical support. If the sample is underpowered or too heterogeneous to detect effects, this should be acknowledged as a limitation rather than reframed as hidden evidence for the hypothesis.

Per your feedback, we more centrally acknowledged the heterogeneity factor as a limitation and tailored language in the discussion section to indicate that the lack of significance should be cautiously interpreted (p. 12).

The Introduction becomes unclear toward the end, particularly in explaining the study's approach and rationale. This section would benefit from revision for clarity and flow.

Thank you for pointing this out. We agree that the end of the introduction could be clarified and streamlined. We hope that the new version is easier to follow (p. 5).

The paper lacks a Conclusions section, which is standard for this journal and would help summarize the key findings and implications.

Per your feedback, we have added a Conclusion section at the end.

The Discussion at times reads as circular, restating assumptions from the Introduction as if they were demonstrated by the results. The authors should more clearly distinguish between what their data show and what prior literature suggests.

Thank you for pointing this out. We’ve updated the Discussion section, making sure to more clearly distinguish prior literature and results from our study. We hope this version is less confusing and circular.

The data availability statement indicates that data sharing is subject to funder approval. This is inconsistent with PLOS's open science requirements and should be addressed.

We thank the reviewer for noting this inconsistency. We agree that the original Data Availability Statement did not adequately align with PLOS Digital Health’s data sharing requirements. We have revised the Data Availability Statement to clarify the conditions under which the data can be accessed. Because the dataset consists of individual-level electronic medical record (EMR) data containing potentially identifiable health information, it cannot be made publicly available. However, access to de-identified data may be granted to qualified researchers upon reasonable request, subject to institutional data use agreements and applicable regulatory and ethical approvals, rather than discretionary funder approval. The revised statement now accurately reflects the data governance constraints while aligning with PLOS policy on controlled-access data. We have removed language suggesting funder-level discretion over data sharing.

The confidence interval reporting in the Abstract is confusing (e.g., "1.8 (0.57, CI: 1.03-3.03)"). Please clarify and ensure consistency with Table 2.

We appreciate your feedback. In the abstract, we report the odds ratio confidence interval. We also updated Table 2 to ensure consistency (now Table 3).
